# Levels of soluble tumor necrosis factor receptor 1 and 2 are associated with survival after ST segment elevation myocardial infarction

**DOI:** 10.1038/s41598-022-18972-5

**Published:** 2022-08-30

**Authors:** Rahel Befekadu, Magnus Grenegård, Anders Larsson, Kjeld Christensen, Sofia Ramström

**Affiliations:** 1 Department of Laboratory Medicine, Section for Clinical Immunology and Transfusion Medicine, Örebro University Hospital, 70185 Örebro, Sweden; 2 Cardiovascular Research Centre, School of Medical Sciences, Faculty of Medicine and Health, Örebro University, Örebro, Sweden; 3 Department of Medical Sciences, Uppsala University, Uppsala, Sweden; 4 Karlstad Central Hospital, Karlstad, Sweden; 5 Department of Cardiology, Örebro University Hospital, Örebro, Sweden; 6 Department of Clinical Chemistry, Department of Biomedical and Clinical Sciences, Linköping University, Linköping, Sweden

**Keywords:** Cardiovascular diseases, Prognostic markers

## Abstract

The soluble tumor necrosis factor receptors (sTNFR1 and sTNFR2) are suggested to play dual roles on physiological and pathophysiological actions of TNF-α. The aim of this study was to investigate the dynamic changes of these biomarkers in patients with ST-segment elevation myocardial infarction (STEMI). Blood was collected from 165 STEMI patients at admission, 1–3 days and 3 months after percutaneous coronary intervention (PCI) and from 40 healthy blood donors. sTNFR1 and sTNFR2 were measured with ELISA. The plasma levels of both sTNFR1 and sTNFR2 were significantly higher than in healthy donors at all three time points. We found no significant differences in sTNFR1 or sTNFR2 when comparing patients with patent versus occluded culprit vessels, or between patients having a thrombus aspiration or not. Survival analysis was performed comparing patients with levels of biomarkers above and below the median values at that time point. We found significant differences in survival for sTNFR2 in acute samples (*p* = 0.0151) and for both sTNFR1 and sTNFR2 in samples 1–3 days after PCI (*p* = 0.0054 and *p* = 0.0003, respectively). Survival analyses suggest that sTNFR1 or sTNFR2 could be promising markers to predict mortality in STEMI patients after PCI.

## Introduction

The inflammatory response has an important role during the acute phase and healing processes after acute myocardial infarction (AMI). Tumor necrosis factor alpha (TNF-α) is produced by several cells such as cardiac myocytes, smooth muscle cells and endothelial cells, and the expression has been shown to be upregulated in myocardial infarction^1^. TNF-α can be considered both a locally acting mediator and a circulating cytokine, as it can be both membrane-associated and proteolytically cleaved to circulating TNF-α. There are two forms of TNF-α receptors (TNFR1) and (TNFR2) involved in TNF-α signaling. The two forms of TNF-α together with the different pathways being activated by the different receptors create a complex picture with sometimes conflicting results being reported. In the cardiovascular system, the overall picture from previous research seems to point towards detrimental effects by signaling through TNFR1 while membrane-bound TNF-α signaling through TNFR2 may be cardioprotective, but the situation is complex and not easily summarized, as both the timing, duration, levels and cell types producing and targeted by TNF-α will influence the outcome (reviewed by^2–4^). In addition, there are indications that both age and sex may influence TNF-α signaling (reviewed by^3^).

Enzymatic cleavage can cause release of soluble TNF-α receptors in plasma (referred to as sTNFR1 and sTNFR2). It has been shown that infusion of TNF-α leads to release of soluble receptors to the circulation^5^. The role of the soluble receptors may be dual, as it has been shown that low levels may help to stabilize and increase availability of TNF-α, while higher levels appear to inhibit the action of TNF-α in in vitro cell growth experiments^6^. Circulating sTNFRs may thus have dual effects on physiological and pathophysiological actions of TNF-α.

In prospective studies, high levels of sTNFRs have been associated with cardiovascular events and increased mortality both in patients with stable coronary artery disease^7,8^, non-calcified atherosclerotic plaque^9^, and diabetes^10^. It has also been associated with increased risk for heart failure^11^, incident myocardial infarction^12^ and cardiovascular death^13^ in population-based studies. Increased levels have also been found in patients with recurrent ventricular arrhythmia^14^ or recurrent myocardial infarction^15^ after coronary stenting. However, only a few studies so far have measured sTNFRs in patients with acute ST-segment elevation myocardial infarction (STEMI)^16–18^. In this study, we aimed to investigate the dynamic changes in sTNFR1 and sTNFR2 in STEMI patients at different time points, before, 1–3 days after and 3 months after a percutaneous coronary intervention (PCI) and to study whether this had any correlation to the long-term mortality of the patients.

## Materials and methods

### Study participants

**Figure 1:**
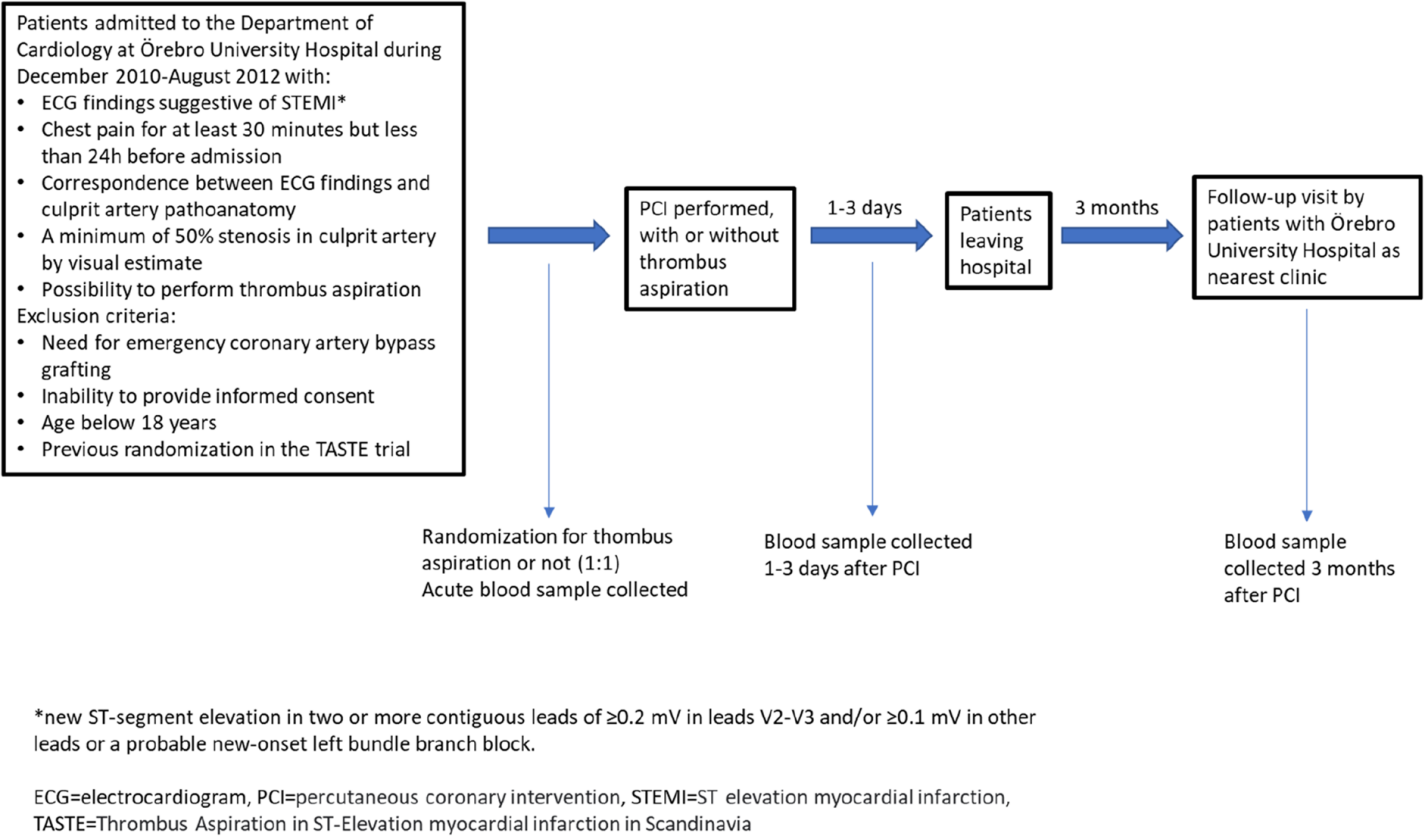
The design of the study.

In this retrospective study, samples were collected from 165 consecutive patients with acute ST elevation myocardial infarction (STEMI) at the Department of Cardiology at Örebro University Hospital from December 2010 to August 2012. The time between symptoms and admission was between 30 min and 24 h. Samples were collected at admission before the percutaneous coronary intervention (PCI) (hereinafter referred to as acute samples), 1–3 days after PCI and 3 months later. Blood samples were collected from the antecubital vein, into 3 mL sodium citrate vacutainer tubes. As this study was conducted as a sub-study of the multicenter TASTE study (Thrombus Aspiration in ST-Elevation myocardial infarction in Scandinavia, ClinicalTrials.gov Identifier: NCT01093404), half of the patients were also randomly assigned for manual thrombosis aspiration in conjunction with the PCI program. The study design and patient selection criteria for the TASTE study have been previously published^19^, and the design of the present sub-study is shown in Fig. 1. As some data was missing for a few participants, each chart and table indicate the exact number of individuals included in each analysis.

The healthy controls were blood donors at the Blood Center at Örebro University Hospital, who agreed to donate an extra blood sample together with their regular donation. They fulfilled the regular health criteria for blood donors, but according to the recruitment regime, they were not specifically selected to match the patients, and gender and age were the only data provided for these donors in agreement with the ethical permit. The mean age was 44 years, and 42% were women. The study was approved by the Regional Ethical Review Board in Uppsala, Dnr 2010/277 and Dnr 2010/294, informed consent was obtained from all subjects and all methods were carried out in accordance with relevant guidelines and regulations.

### Laboratory analysis

The collected venous blood samples were centrifuged at 2000 × g for 10 min prior to laboratory testing. The plasma was divided into aliquots and stored at − 80 °C for analyses that were not immediately performed.

sTNFR1 and sTNFR2 were analyzed by sandwich ELISAs from R&D Systems (Minneapolis, MN, USA, products DY225 and DY726, respectively) according to the manufacturer’s instructions, and the absorbance was measured in a Spectra Max 250 (Molecular Devices, Sunnyvale, CA, USA). The ELISAs had total CVs of approximately 6%. All assays were performed blinded to clinical information. For statistical analysis, values below the limit of quantification were entered as the lowest concentration within the range of quantification.

### Statistical analysis

GraphPad Prism 8 (GraphPad Software Inc., San Diego, CA, USA) was used for statistical analysis. For unpaired data, the non-parametric Kruskal Wallis test was performed and for paired data, the non-parametric Friedman test was used, followed by Dunn´s multiple comparison test for post-hoc comparisons. *P* < 0.05 was considered as statistically significant. Correlation analysis was performed using Spearman correlation for non-parametric data. The Log-rank test (Mantel-Cox method) was performed for comparison of survival curves.

## Results

### Baseline demographics and clinical characteristics

We studied 165 patients with mean age 69 years (range 43–94) admitted with STEMI to the emergency department between December 2010 and August 2012. Of the 165 patients, 118 (76 males and 28 females**)** were still alive and 47 (29 males and 18 females**)** had died at the follow up analysis in March 2019. Of the enrolled patients, 17% had experienced a previous myocardial infarction, and 34% were active smokers. The mean body mass index (BMI) was 27.1 kg/m^2^ with a range of 16.3–39.1. According to the patient anamnesis, 52% had hyperlipidemia, 46% hypertension, 18% diabetes mellitus and 10% unstable angina. The platelet particle concentration was 228 ± 62 × 10^3^/µL (mean ± SD), and the white blood cell count was 10.8 ± 4.1 × 10^3^/µL (mean ± SD). At admission before PCI, 22% of the patients were taking acetylsalicylic acid (ASA) only, 2% were on ASA + clopidogrel, 27% were on betablockers and 26% were on statins.

### Differences in sTNFR1 and sTNFR2 between patients and healthy donors

**Figure 2:**
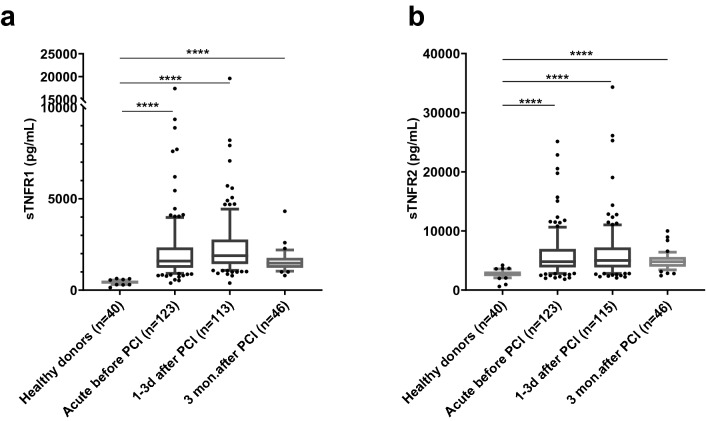
Plasma levels of **(a)** sTNFR1 **(b)** sTNFR2 in blood samples obtained from STEMI patients before, 1–3 days after PCI and 3 months after PCI as compared to healthy donors. Statistical analyses were conducted using Kruskal Wallis test followed by Dunn´s multiple comparison test, the whiskers show the 10–90th percentile with all outliers. (NS = not significant, **** = *p* < 0.0001).

When comparing the levels of sTNFR1 and sTNFR2 in samples from the STEMI patients with samples from healthy donors, both biomarkers showed plasma levels significantly higher than the healthy controls at all three time points (Fig. 2a, b).

During PCI, the patients were categorized into two groups, one including patients with patent (partially open) culprit vessel, while the other group had a totally occluded culprit vessel before PCI. Table 1 also shows the data divided into these categories. However, no significant differences were found for any of the time points investigated. Similarly, no significant differences were found between patients who were randomized to having thrombus aspiration or not.

**Table 1:** Quantitative data on levels of sTNFR1 and sTNFR2 in the study population and the healthy donors. Data are displayed as median (lower level of 95% confidence interval, upper level of 95% confidence interval, n).

	With patent culprit vessel	With occluded culprit vessel	With thrombus aspiration	No thrombus aspiration	All
**sTNFR1 (pg/mL)**					
Acute	1455 (1250, 1760, n = 38)	1689 (1439, 2050, n = 82)	1527 (1369, 1898, n = 62)	1668 (1464, 1898, n = 57	1597 (1439, 1760, n = 123)
1–3 days after PCI	1680 (1487, 1969, n = 33)	2006 (1795, 2361, n = 75)	1760 (1527, 2250, n = 54)	1977 (1772, 2406, n = 53)	1886 (1680, 2198, n = 113)
3 months after PCI	1560 (1240, 1760, n = 19)	1480 (1160, 1920, n = 24)	1560 (1160, 1800, n = 23)	1480 (1280, 1720, n = 20)	1480 (1360, 1720, n = 46)
Healthy donors	–	–	–	–	453 (401, 491, n = 40)
**sTNFR2 (pg/mL)**					
Acute	4666 (4126, 5783, n = 38)	4910 (4558, 5814, n = 82)	4584 (4338, 5551, n = 62)	5106 (4470, 6050, n = 57)	4803 (4540, 5425, n = 123)
1–3 days after PCI	4760 (3652, 5868 n = 33)	5054 (4693, 6186, n = 77)	4876 (4217, 5366, n = 56)	5197 (4692, 6607, n = 53)	5011 (4692, 5607, n = 115)
3 months after PCI	4760 (4080, 5400, n = 19)	4860 (3640, 5840, n = 24)	4760 (3680, 5800, n = 23)	4960 (4080, 5400, n = 20)	4800 (4240, 5280, n = 46)
Healthy donors	–	–	–	–	2651 (2484, 2923, n = 40)

### Changes in sTNFR1 and sTNFR2 at different time points

Since we did not observe any significant differences between individuals with patent and occluded culprit vessels, we analyzed these together in the upcoming analysis of changes in levels of sTNFR1 and sTNFR2 at the different time points. To avoid inter-individual variance to affect the results, we only included patients available for sampling at all three time points and used paired analysis for the statistical testing.

**Figure 3:**
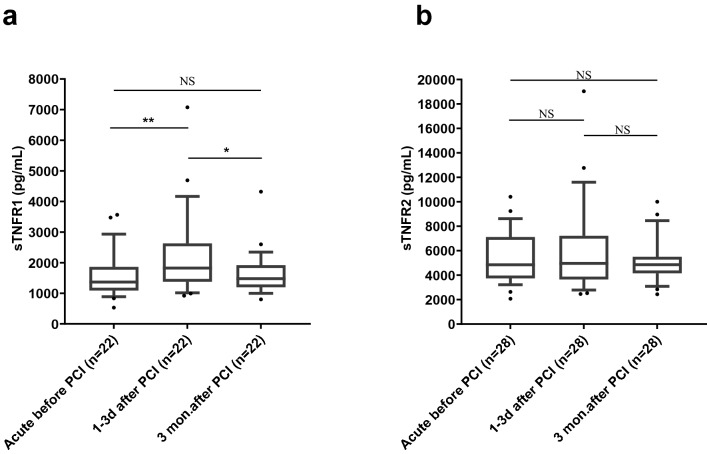
Plasma levels of **(a)** sTNFR1 and **(b)** sTNFR2 in paired samples obtained at different time points during STEMI. Statistical analyses were conducted using the Friedman test followed by Dunn´s multiple comparison test, and data are shown as median and 10–90th percentile with all outliers. ((NS = not significant, * = *p*  < 0.05, ** = *p* < 0.01).

For sTNFR1, there was no significant difference between the plasma levels in the acute samples and the three months after PCI samples, but in the sample 1–3 days after PCI, the sTNFR1 plasma levels were significantly higher than both the other two time points (Fig. 3a). For sTNFR2, the increased levels were relatively stable throughout the observation period, as there were no significant differences in levels between any of the three time points (Fig. 3b).

### Correlation between sTNFR1, sTNFR2 and troponin at different time points

**Figure 4:**
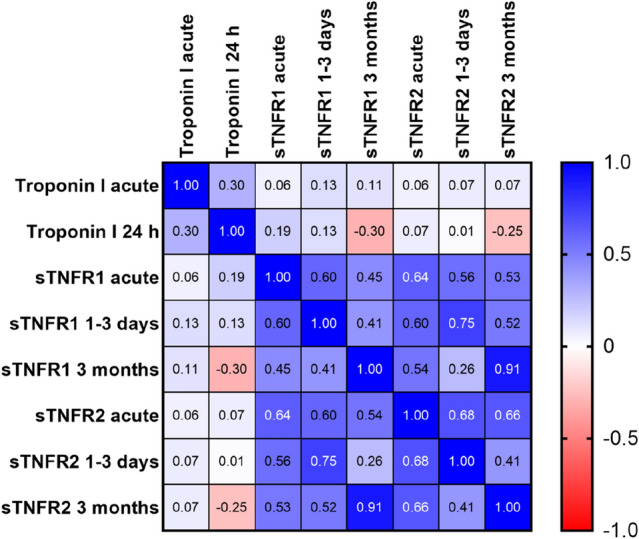
Correlation matrix showing Spearman r values for correlations between levels of sTNFR1 and sTNFR2 at the different timepoints.

Figure 4 shows the Spearman correlation coefficients between sTNFR1, sTNFR2 and troponin at different time points. For sTNFR1, r was 0.60 and 0.45 when comparing acute values to the ones at 1–3 days and 3 months, respectively. For sTNFR2, r was 0.68 between acute and 1–3 days, and 0.66 between acute and 3 months. The correlations between sTNFR1 and sTNFR2 at the same time points were strong. In acute samples, r was 0.64. At 1–3 days, r was 0.75, and the strongest correlation between these markers were at 3 months, where r was 0.91. In these patients, troponin I was measured both in acute samples and after 24 h, but none of the markers showed strong correlations to troponin I at any of these time points.

### Survival analysis of patients with elevated levels of sTNFR1 and sTNFR2

**Figure 5:**
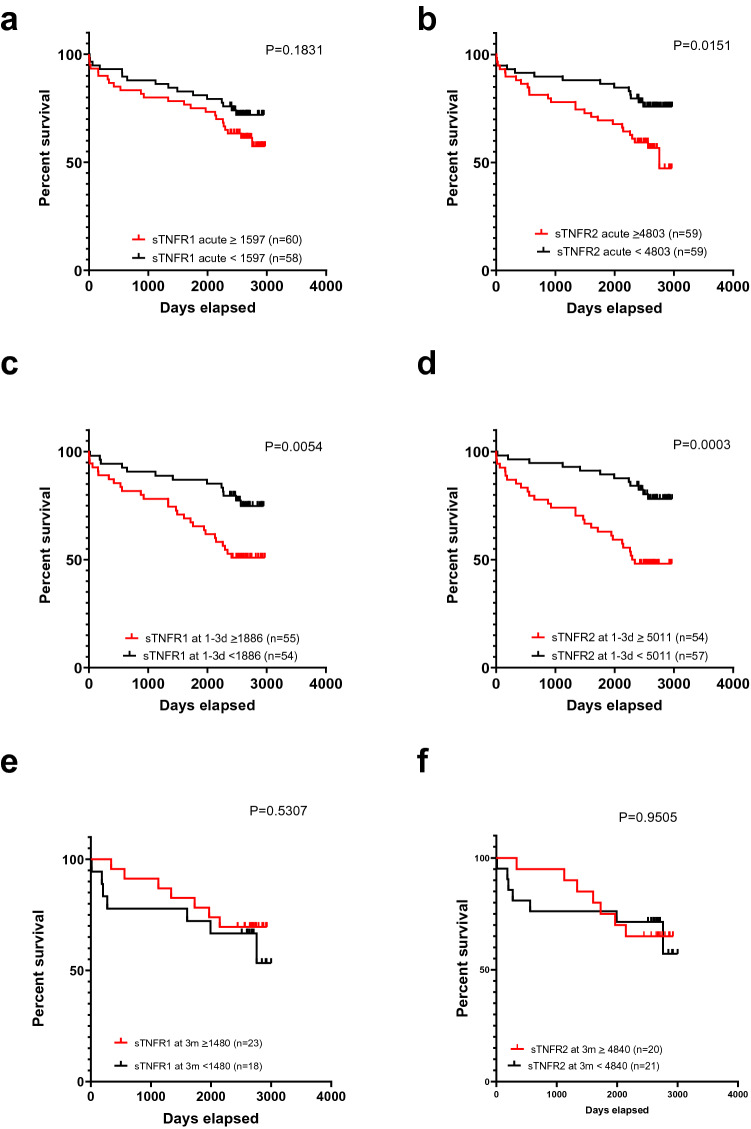
Kaplan–Meier curves comparing survival for patients with levels above and below the median value for **(a, c, e)** sTNFR1 **(b, d, f)** sTNFR2. Curves in **(a)** and **(b)** are for acute samples, while **(c)** and **(d)** are for samples collected 1–3 days after PCI and **(e)** and **(f)** are for samples collected 3 months after PCI. Survival analysis was performed using the log-rank test (Mantel-Cox method).

The number of days from inclusion to death (or days from inclusion to extraction of data from patients who were still alive at the end of the study) was calculated to determine if levels of sTNFR1 or sTNFR2 were associated with patient survival. Patients were then divided into groups with higher or lower levels of sTNFR1 or sTNFR2 than the median value at the same time point. Kaplan–Meier curves were generated from this data and are shown in Fig. 5. The survival of the groups was compared using the log-rank (Mantel-Cox) test.

**Figure 6:**
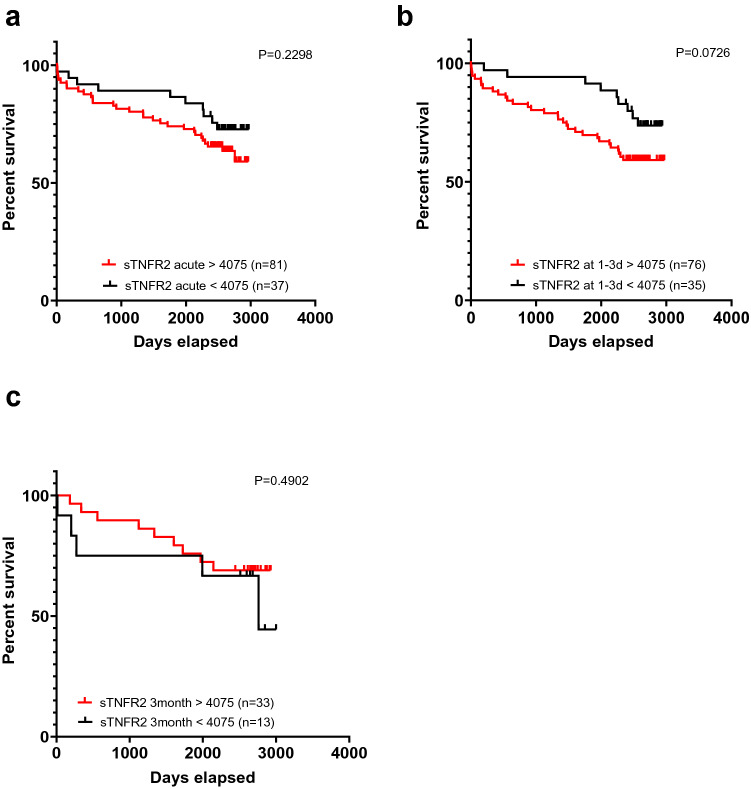
Kaplan–Meier curves comparing survival for patients with levels above and within the reference range for the 40 healthy donors for sTNFR2 in **(a)** acute samples and **(b)** samples collected 1–3 days after PCI and **(c)** samples collected 3 months after PCI. Survival analysis was performed using the log-rank test (Mantel-Cox method).

As shown in Fig. 5, significant differences in survival were observed for sTNFR2 in acute sample and for both sTNFR1 and sTNFR2 in samples collected 1–3 days after PCI. We also tested making survival plots with the cutoff at the upper reference limit (mean + 2SD) calculated from our 40 healthy blood donors, but as very few patients were below this limit for sTNFR1 (n = 3 in acute samples, n = 1 after 1–3 days and n = 0 after 3 months), this was not a feasible approach for this marker. For sTNFR2, the groups were more evenly sized, but as shown in Fig. 6, no significant differences were observed using this approach.

## Discussion

In this study, we analyzed levels of sTNFR1 and sTNFR2 in samples collected from STEMI patients before (acute sample), 1–3 days after and 3 months after PCI. We found that the levels of sTNFRs were significantly elevated at all three time points as compared to our healthy donors. Only one of the other published studies in STEMI patients^17^ did include a control group. When comparing our data to theirs, our control group was younger and with more female donors, and we observed lower levels of sTNFR1 but higher levels of sTNFR2, but in both studies, the sTNFR levels in the patients were significantly higher than in the control group. However, it has to be noted that the levels of sTNFR1 reported by us and others^16–18^ in STEMI patients are relatively similar to the ones reported in prospective studies in patients with stable CAD^7,8,20^, and also to levels reported in population-based studies in older adults^9,11–13^. Thus, the level of sTNFR1 may not be of diagnostic interest on the population level in this age group. On the other hand, similar to us, both Vaglimigli et al^17^ and Paccalet et al^16^ report an increased risk of adverse events in STEMI patients with higher sTNFR1 levels, and Nilsson et al^18^ shows associations between levels of sTNFR1 and the final infarct size. Also, the two studies with measurements at different time points^16,18^ show the same trend as us with increasing levels of sTNFR1 between admission and 24 h, which might indicate a rapid response to the acute event and following intervention which is mirrored in the levels of sTNFR1 released. Interestingly, the only time point where the levels of sTNFR1 showed prognostic value in our data was in the sample collected 1–3 days after PCI, if this indicates that the immediate response after the PCI procedure is the most prognostic may be an area for future studies.

For sTNFR2, only one study report values as high as ours^8^, while the other studies that included this marker^7,11,12,16–18,20^ report levels more similar to the ones in our control group. The reason for this is unclear, all these studies have used ELISA kits from the same manufacturer, and even though analyses have been performed in plasma in some^12,13,18,20^ and in serum in others^10,12,16,17^ this did not seem to have created any consistent differences in levels. Also, we did not observe any differences in levels between the investigated time points, while Nilsson et al^18^ report increased levels at 24 h and Paccalet et al. shows increasing levels up to 48 h followed by a decrease towards admission levels after one month^16^. One possible reason for us not observing any differences is that only a fraction of the 165 patients were investigated at all three time points, as the others were from other regions and thus doing their follow-up visit at 3 months elsewhere. This reduces the strength of the data, but we still believe it is more correct to do so, considering the high variability between individuals. Regardless of this, we did observe a decreased survival for the patients with the highest levels of sTNFR2, both for acute samples and samples collected 1–3 days after PCI. This supports other studies reporting sTNFR2 as a prognostic marker for cardiovascular disease or mortality^16,18,20^, while there are a few studies measuring both markers that only finds sTNFR1 to be predictive^11,17^.

When looking at the proposed mechanisms and signaling pathways activated by the different TNF receptors, it may seem surprising that high levels of sTNFR2 should predict worse outcome. But it is important to keep in mind that what is measured here is the levels of the soluble receptors, which according to the findings by Lantz et al^5^ may be a reaction to increased levels of TNF-α in the system, as they showed that infusion of TNF-α leads to release of soluble receptors to the circulation. In support for this hypothesis is the high correlation between the levels of both receptors, which is seen both in our data and in other studies^12,20^. As TNF-α is released upon plaque rupture^21^, measurements of TNF-α could be assumed to be a more accurate way of studying the physiological processes, but this was not seen as a good alternative, as TNF-α has been reported to have a short half-life in stored samples^16^. However, studies measuring both TNF-α and sTNFRs show similar trends for these markers^14,15,20^, which makes it likely that we would have seen the same results also with TNF-α in our patients.

In our study, none of the soluble receptors showed any correlation to the levels of troponin I. In other studies, conflicting data regarding this has been reported, there are both studies reporting a correlation^18^, and those reporting that the levels of circulating sTNFR1 and sTNFR2 were “independent of cardiac enzyme release”^16^. In our previous study^22^, we showed that the levels of troponin I were significantly higher in patients with occluded culprit vessels, but no differences between patients with occluded and partially open culprit vessels or between patients with and without thrombus aspiration were observed for the sTNFRs.

## Conclusion

We conclude that high levels of sTNFR1 and sTNFR2 were associated with mortality in our study population. However, the results in our population may not be generalizable to groups with other demographic parameters. If levels of sTNFRs are to be discussed as prognostic markers, it has to be noted that there might be an increase with age, and that other conditions also affect the levels, with higher levels reported in patients with diabetes type 2^10^ and much lower values (lower than for healthy donors) in patients with chronic heart failure^23,24^. In addition, kidney function influences the levels, also in patients with cardiovascular disease^20^. Thus, a bigger sample size and thorough knowledge of other characteristics of the patient group, also including e.g. ventricular ejection fraction, area of blood supply for the symptom-dependent coronary artery and response to given therapeutic treatment is recommended for future studies in order to fully investigate the potential of these markers as predictors of outcome in STEMI patients.

## Data Availability

The datasets for the current study are available from the corresponding author on request. Depending on the extent of the request, this may also require an additional ethical permit.

## Abbreviations

AMI: Acute myocardial infarction
ASA: Acetylsalicylic acid
BMI: Body mass index
CV: Coefficient of variation
ELISA: Enzyme linked immunosorbent assay
MI: Myocardial infarction
NS: Not significant
PCI: Percutaneous coronary intervention
STEMI: ST-elevation myocardial infarction
sTNFR1: Soluble tumor necrosis factor receptor 1
sTNFR2: Soluble tumor necrosis factor receptor 2
TASTE: Thrombus Aspiration in ST-Elevation myocardial infarction in Scandinavia
TNF-α: Tumor necrosis factor-alpha
